# Field Calibration of Wind Direction Sensor to the True North and Its Application to the Daegwanryung Wind Turbine Test Sites

**DOI:** 10.3390/s8127782

**Published:** 2008-12-03

**Authors:** Jeong Wan Lee

**Affiliations:** Division of Mechanical Engineering and Mechatronics, Kangwon National University, 192-1, Hyoja2-dong, Chunchon, Kangwon-do, 200-701, South Korea; E-Mail: jwlee@kangwon.ac.kr; Tel.: +82-33-250-6377; Fax: +82-33-257-4190

**Keywords:** Field Calibration, Wind Direction Sensor, Image Analysis and Processing, Uncertainty Analysis

## Abstract

This paper proposes a field calibration technique for aligning a wind direction sensor to the true north. The proposed technique uses the synchronized measurements of captured images by a camera, and the output voltage of a wind direction sensor. The true wind direction was evaluated through image processing techniques using the captured picture of the sensor with the least square sense. Then, the evaluated true value was compared with the measured output voltage of the sensor. This technique solves the discordance problem of the wind direction sensor in the process of installing meteorological mast. For this proposed technique, some uncertainty analyses are presented and the calibration accuracy is discussed. Finally, the proposed technique was applied to the real meteorological mast at the Daegwanryung test site, and the statistical analysis of the experimental testing estimated the values of stable misalignment and uncertainty level. In a strict sense, it is confirmed that the error range of the misalignment from the exact north could be expected to decrease within the credibility level.

## 1. Introduction

It is very important to correctly measure the wind when setting up the right research direction for the utilization of wind energy. The same is vital for the correct evaluation of wind resources at the particular site. When a site calibration of a wind turbine test site is conducted, an inaccuracy in positioning the wind direction sensor can cause severe errors in calculating the calibration relationship of the wind energy between the reference location and the wind turbine installation location [1, 2].

There are generally two factors causing the error between the real wind direction and the measured wind direction. The first factor is the error caused by the non-linearity of potentiometer inside the wind direction sensor. The second is the error caused by a discord with the true north that happens during the installation of the meteorological mast (MM). The first error can be corrected within the allowed tolerance through the sensor calibration in the laboratory [3]. The second error, however, can only be minimized by aligning the wind direction sensor of the MM to the true north at the time of the installation of the MM [4, 5].

**Figure 1.**
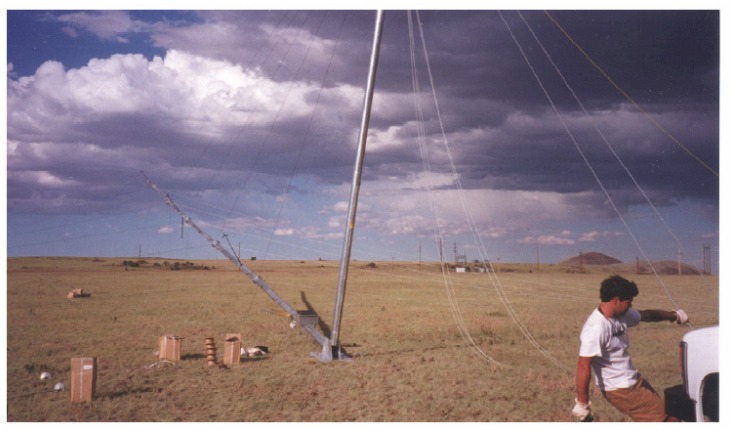
Meteorological mast erection using gin pole and winch.

The wind direction sensor's accurate alignment of the north direction to the true north is not an easy job, particularly in circumstances where the MM is being installed. Great difficulties arise, particularly when the MM is built very high. In the Daegwanryung wind turbine test site, the height of the MM is 46 m. The installation of the long MM was accomplished by assembling its components on the ground, and then erecting the assembled mast from the floor (Figure 1). Although the exact alignment of the mast to the true north was done on the ground, the discordance with the exact north occurred during the process of erection and construction. Furthermore, the high elevation of the MM causes torsion, which generates the misalignment to the true north [6, 7].

In this study, I propose a calibration technique to align to the true north to solve the discordance problem of the wind direction sensor. The misalignment of the tower to the exact north was corrected by measuring the accurate course of the wind direction sensor using the image taken by a camera, and simultaneously comparing it with the voltage value from the wind direction sensor. The blade section of the wind direction sensor from the image signal was separated by the segmentation method, and then the deviation degree was calculated by applying the least squares error method to the segmented image signal.

We also performed an uncertainty analysis on the component error for the suggested method in a practical situation. An experiment applying the method on the MM at the Daegwanryung wind turbine test site was conducted and the validity of the method was confirmed.

In the following sections the experimental setup of the proposed technique will be presented. Section 3 will present the process of evaluating the true wind direction from the camera image captured. In Section 4, some uncertainties and the accuracy of the proposed technique will be analyzed. This will be followed by the research conclusion in Section 5.

## 2. Experimental Setup

**Figure 2.**
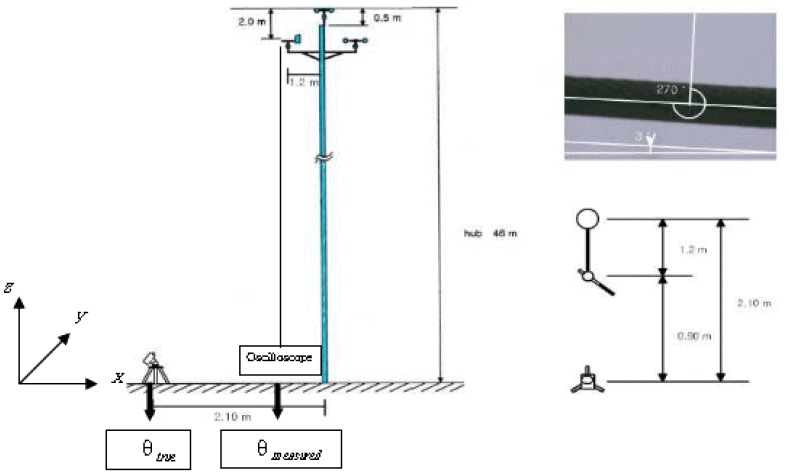
Schematic diagram of the experimental setup.

The organizational diagram of the total system, which was used at the Daegwanryung wind turbine test site, is shown in Figure 2. The basic principle of this technique is as follows: the real wind direction value, *θ_true_* was evaluated by the measurement of the rotation direction of the wind direction sensor using the camera at a certain time. At the same time the measured direction value, *θ_measured_* was measured by the output voltage from the sensor. Finally the degree of misalignment to the true north was calculated by comparing the value of *θ_true_* and *θ_measured_*.

Ideally, the *x* and *y* positions of the camera, (*x*, *y*)*_camera_* should be identical with that of the wind direction sensor (*x*, *y*)*_sensor_*. That is, the camera should be located in a straight line with the wind direction sensor for *z* direction. In a real situation, however, the photograph of the wind direction meter cannot be taken at the ideal location because the peripheral devices attached to the wind measuring tower hinder the view. To overcome this problem, the camera was built horizontally at 0.9 m below the ideal location.

Meanwhile, the determination of the absolute rotation position of the exact north for the *z* direction of the installed camera was needed. Thus, the true north direction was measured using the global positioning system (GPS) and compass. The upper right side photograph in Figure 2 indicates the absolute rotation position of the camera about the true north.

The camera employed in the experiment was a combined digital camera and camcorder by SONY, and the photograph was taken using the maximum zoom, 20 magnifications, and 640 × 480 pixel mode. The wind direction sensor used in this experiment was NRG #220P and the specifications of the sensor are described in Table 1 [8].

The measurement of *θ_merasured_* was achieved by recording the output voltage of the wind direction sensor with the digital oscilloscope, Tektronics TS220. The trigger of the photographing moment and the output voltage measurement was initiated by the use of the RUN/STOP function in the digital oscilloscope.

## 3. Determination of *θ_true_* through Image Processing

**Figure 3.**
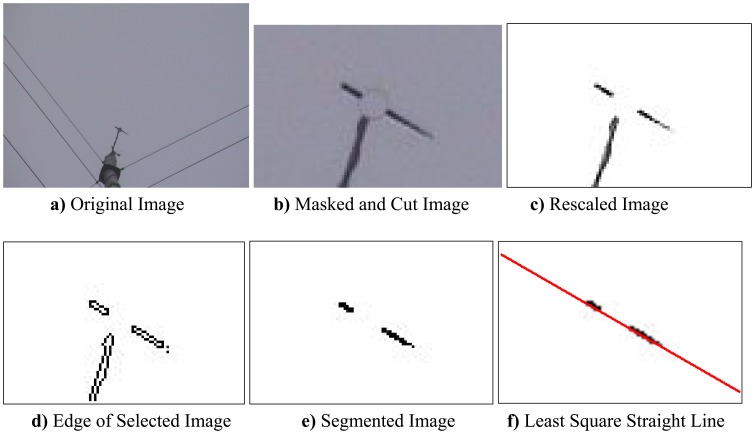
Procedure of image data analysis to calculate *θ_true_*.

The true angle of the wind direction sensor, *θ_true_*, was calculated through the image signal processing shown in Figure 3. Figure 3(a) is an example of an original image captured during the experiment. Since the height of the wind measuring tower at the Daegwanryung test site is very high (hub height is 46 m), the image area of the sensor blade section occupied a small part on the total area of the photograph despite the maximum zoom capability of the camera.

The overall image processing begins when the blade part of the wind direction sensor from the original image is divided by the segmentation method; the slope of the first-order function based on the (*x*, *y*) data of the parted image area was then obtained using the least square error method. Finally, the deviation degree from the true north was evaluated.

This process was implemented with the image processing tool box function in MATLAB [9]. The overall process was accomplished through the following steps: First step was to make an effective cut image of the pertinent region, which included cutting the wind direction sensor in the original image and then removing the untouched region of the sensor through masking [Figure 3(b)]. In the second step, the dynamic range of the white and black of the image signal was widened by utilizing the threshold. Three objects were shown in Figure 3(c), but only two objects could be seen as a blade of wind direction sensor. In the third step, the boundary curve of the image was obtained [Figure 3(d)] by using the gradient method. This step used edge and Sobel operator to calculate a binary mask that contains the segmented objects.

In the fourth step, the segment that was connected to the border of the image could be removed, leaving only the desired segments for the sensor blade section, and then the interior gaps were filled by using fill function [Figure 3(e)]. In the final step, the slope of the sensor blade was indicated by the least square error method on the basis of the segmented value.

## 4. Uncertainty Analysis

For this section, we analyze the uncertainty that can occur when measuring the wind direction with the proposed technique. This analysis shows that the considered uncertainty was caused by errors in the camera installation position and the camera resolution.

### 4.1. Uncertainty from the error of camera installation position

**Figure 4.**
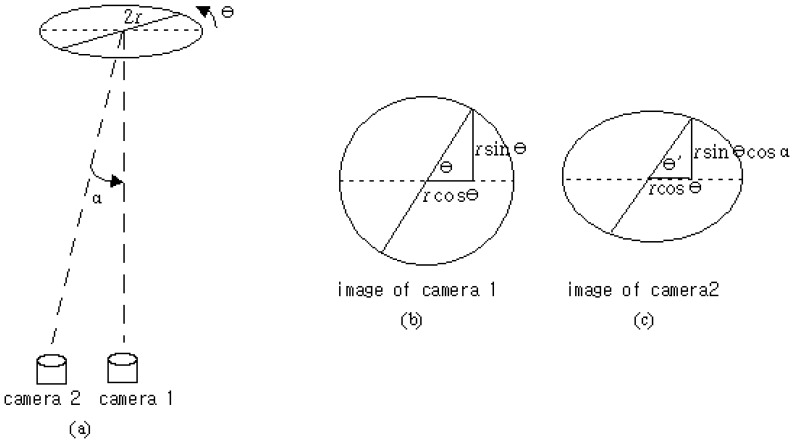
Images from two cameras with different installed positions.

The ideal position of the camera is on the vertical direction from the position (*x*, *y*) of the wind direction meter. However, the blade of the wind direction meter cannot be captured at the exact vertical position because of obstructions from the other sensor and the peripheral device attached to the MM. Hence, the deviation of the camera position from the correct vertical direction of the wind direction sensor is inevitable. The measurement uncertainty analysis caused by the camera installation position was carried out as shown in Figure 4.

**Figure 5.**
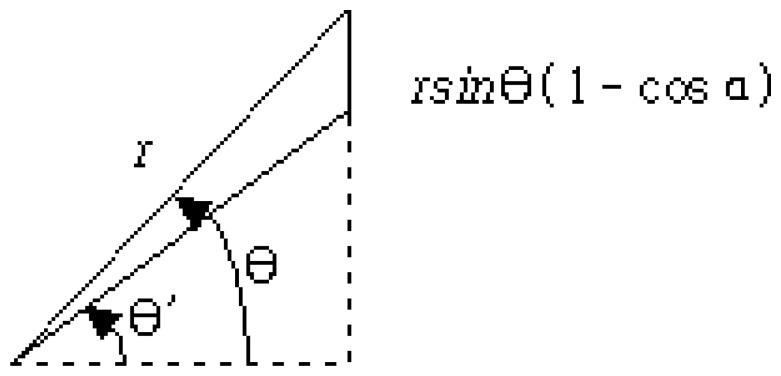
Angle difference of the two cameras.

In Figure 4(a), if the segment of the 2*r* line with the *θ* degree slope at the perfect location is photographed, it will generate an image like the one shown in Figure 4(b). If the photograph at the *α* degree deviation location from the exact location is taken, the captured image will be like Figure 4(c). Figure 5 represents the geometrical difference of *θ* and *θ*'. The difference of the degrees means the amount of the uncertainty caused by the camera position.

The difference between *θ* and *θ*', *θ*_1_ = *θ* − *θ*' as shown in Figure 5 has an inequality relationship represented by equation (1).

(1) $$\begin{array}{l} \begin{array}{ll} \sin(\theta -\theta^{\prime })\leq \frac{r\sin\theta (1-\cos\alpha )}{r}=\sin\theta (1-\cos\alpha ), & 0\leq \theta \leq \pi /2 \end{array}\\ \begin{array}{ll} \theta_{1}=\theta -\theta^{\prime }\leq \sin^{-1}[\sin\theta (1-\cos\alpha )], & 0\leq \theta \leq \pi /2 \end{array} \end{array}$$

The maximum value of *θ*_1_, which will be the greatest error caused by the uncertainty of camera location, can be obtained at *θ* = 90°. Equation (2) represents the maximum value of *θ*_1_.

(2) $$\begin{array}{cc} \theta_{1\max}\leq \sin^{-1}(1-\cos\alpha ), & 0\leq \alpha \leq \pi /2 \end{array}$$

### 4.2. Uncertainty from the error of camera image resolution

The accuracy of *θ_true_* which is estimated form the captured image of camera depends on the resolution accuracy of the camera. Specifically, the pixels consisted in the cut image of blade [Figure 3(b)] are dominant factor. So, the limited resolution of the camera gives rise to uncertainty that induces the calculation error of *θ_true_*. In this subsection, the above uncertainty arising from the camera image resolution is analyzed.

**Figure 6.**
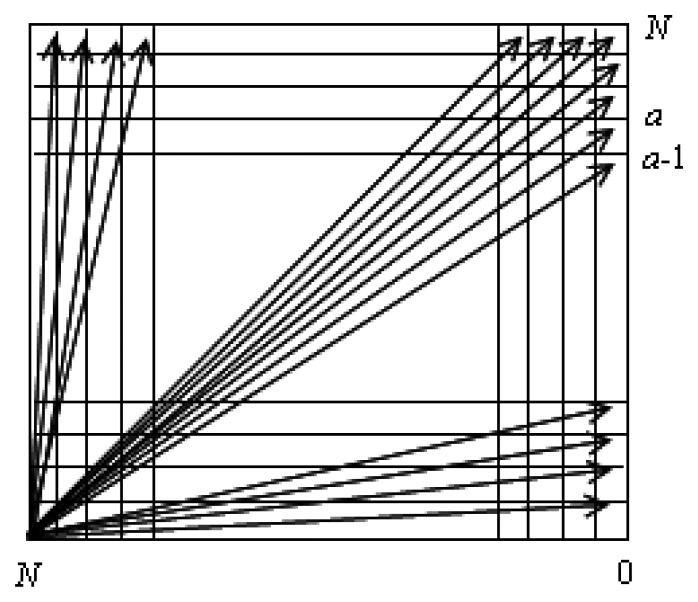
Representative angles in the image of *N* × *N* pixel format.

If the pixel number of the sensor blade length is *N* when the photograph of the sensor blade is taken, the examples of allowable degree for the photographed image is shown in Figure 6 where *N* denotes the number of pixel. A close look at Figure 6 shows that the resolution of the degree at *a*-th pixel position expressed on the image of the *N* × *N* pixel format can approximately be represented as equation 3:

(3) $$\theta_{\text{resolution}}=\tan^{-1}(\frac{\alpha }{N})-\tan^{-1}(\frac{\alpha -1}{N})$$

From equation (3), the maximum value of the uncertainty from the camera resolution occurs as *a* =1. Therefore, the maximum values of *θ*_2_ can be expressed as equation (4):

(4) $$\theta_{2\max}={\left(\theta_{\text{resolution}}\right)}_{\max}\approx \tan^{-1}(\frac{1}{N})$$

## 5. Experimental Results

**Figure 7.**
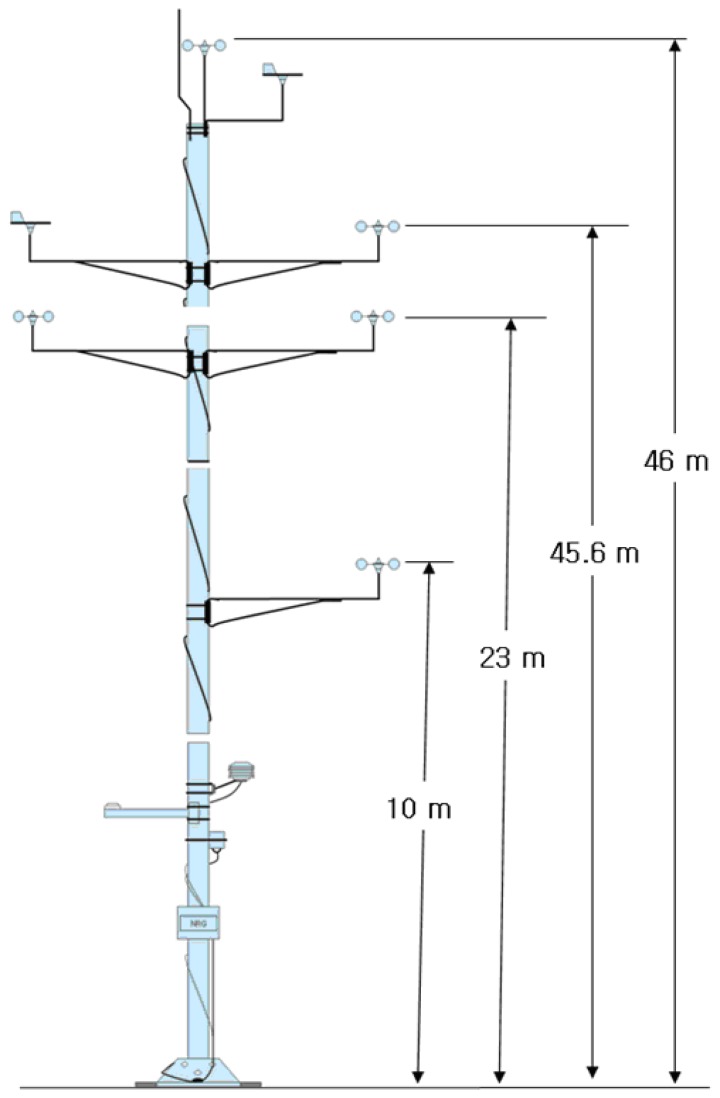
Meteorological mast installed at the Dawanryung test site.

The suggested technique was implemented on the MM at the Daegwanryung wind turbine test site. The height of the MM at the test site was 46 m. Five wind speed meters, two wind direction meters, temperature sensor, pressure sensor, and radiant heat sensor were built on the MM. The MM, schematically shown in Figure 7, was required for the site calibration of wind energy. The proposed technique was applied on the wind direction sensor at the highest point of the tower in the present study.

The position of the camera was installed at point 0.9 meter below the wind direction sensor. Image capture was performed 36 times in 640 × 380 pixel format. The blade section of the wind direction sensor in the captured image was measured with the resolution of 43 × 43 pixels.

The calculated amounts of the uncertainty for the measurement list are shown in Table 2. Here, the uncertainty level caused by the camera position may be almost negligible, but the uncertainty level resulting from the camera resolution was relatively high. The higher uncertainty can be reduced by using a camera with better resolution.

**Figure 8.**
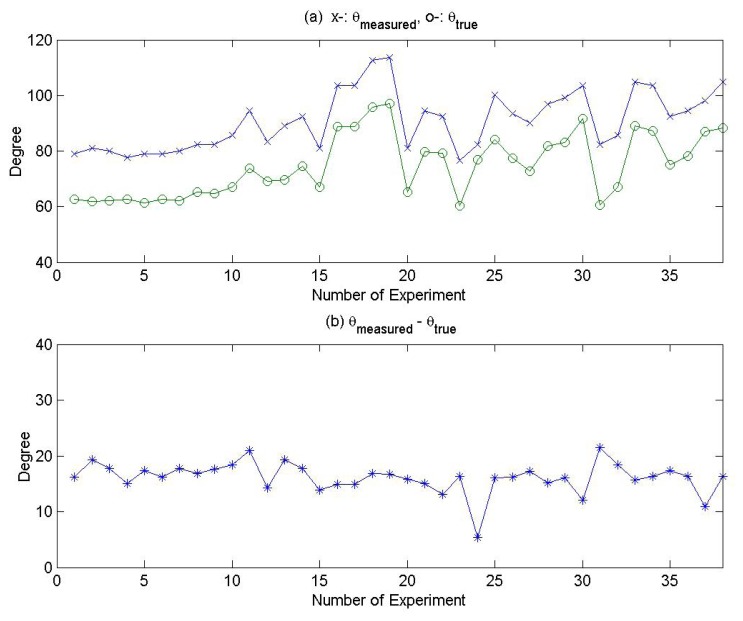
Experimental Result of *θ_true_* and *θ_measured_*.

The value of *θ_true_* and the value of *θ_measured_* from the 36 experiments are plotted in Figure 8(a), and the value of *θ_measured_* − *θ_true_* is plotted in Figure 8(b). Stable offset values between *θ_true_* and *θ_measured_* along the number of experiments are shown in Figure 8(a). The statistical results of *θ_measured_* − *θ_true_* are shown in Table 3. The stable average value comes mainly from the misalignment of MM to the true north in the mast installation, and the standard deviation consists of uncertainties in measuring *θ_true_* and *θ_measured_*. In real applications, all the data of wind energy resources related to wind direction should be compensated based on the average value.

Meanwhile, there was a signal noise in the output voltage of the wind direction sensor (potentiometer type sensor) at the time of measurement. The signal noise can be regarded as a major contributor to the standard deviation in the statistical results. Considering the statistical treatment result and the error analysis of uncertainty, the established wind direction sensor was off by 16.14 degrees ± 3.46 degrees to the true north within 68% credibility level limit. If the resolution of the camera could be improved, and the signal noise in the output voltage eliminated through a filtering technique, the error range of the misalignment from the exact north could be expected to decrease within the credibility level.

## 6. Concluding Remarks

This research proposes a technique which can properly determine through the use of a camera the degree of misalignment of the wind direction sensor installed on the MM, to the correct north. The validity of the technique was affirmed after it was implemented on the MM at the Daegwanryung test site. A quantitative analysis about the uncertainty in the measurement was also conducted. Because the degree of misalignment of the wind direction sensor to the true north can be easily obtained as shown in the present study, it is inferred that the proposed technique can be applied effectively to MMs in other practical situations.

## Figures and Tables

**Table 1.** Specifications for the NRG #220P wind direction vane.

Output Signal	DC voltage from a conductive plastic potentiometer

Resistance	10 KΩ(± 20%)
Deadband	8 degrees
Nonlinearity	1.0% maximum (0.5% typical)
Excitation Voltage	1 to 15 VDC
Sensitivity	Approximately 1 m/s

**Table 2.** Uncertainties in the experiment.

Uncertainty from camera position, *θ*_1max_	0.011 degree
Uncertainty from camera resolution, *θ*_2max_	1.33 degrees

**Table 3.** Statistical results in the experiment.

Average of *θ_measured_* − *θ_true_*	16.14 degree
Standard deviation of *θ_measured_* − *θ_true_*	2.129 degrees

## References

[1] (1999). IEC61400-1. Wind Turbine Generator Systems Part I, Safety Requirement.

[2] (1999). IEC61400-12. Wind Turbine Generator Systems Part II, Wind Turbine Power Performance Testing.

[3] Doeblin E. O. (1989). Measurement Systems, Application and Design.

[4] Kristensen L., Jensen G., Hansen A., Kirkegaard P. (2001). Field Calibration of Cup Anemometers.

[5] Curvers A. (1999). Calibration at the ECN Test Site.

[6] Ferreira M., Rodirigues A., Ribeiro L. (1999). Evaluation of the Influence of the Installation of a Wind Turbine Over the Results of a Nearby Measuring Station.

[7] Hau E. (2000). Wind Turbines Fundamentals, Technologies, Application and Economics.

[8] NRG Systems, Inc. (1999). 9300SA Stand Alone Logger: Customer Guide..

[9] Gonzalez R., Woods R., Eddins S. (2003). Digital Image Processing Using MATLAB.

